# Emerging target discovery and drug repurposing opportunities in chordoma

**DOI:** 10.3389/fonc.2022.1009193

**Published:** 2022-10-27

**Authors:** Daniel M. Freed, Josh Sommer, Nindo Punturi

**Affiliations:** Chordoma Foundation, Durham, NC, United States

**Keywords:** chordoma, rare cancer, drug repurposing, target discovery, multi-omics, functional genomics, synthetic lethality, precision oncology

## Abstract

The development of effective and personalized treatment options for patients with rare cancers like chordoma is hampered by numerous challenges. Biomarker-guided repurposing of therapies approved in other indications remains the fastest path to redefining the treatment paradigm, but chordoma’s low mutation burden limits the impact of genomics in target discovery and precision oncology efforts. As our knowledge of oncogenic mechanisms across various malignancies has matured, it’s become increasingly clear that numerous properties of tumors transcend their genomes – leading to new and uncharted frontiers of therapeutic opportunity. In this review, we discuss how the implementation of cutting-edge tools and approaches is opening new windows into chordoma’s vulnerabilities. We also note how a convergence of emerging observations in chordoma and other cancers is leading to the identification and evaluation of new therapeutic hypotheses for this rare cancer.

## Introduction

Chordoma is an ultra-rare bone cancer that arises in the skull base or spine of pediatrics and adults, and originates from vestigial remnants of the embryonic notochord. Normally a low-grade but locally invasive disease, current standard of care for chordoma involves maximum surgical resection and/or radiotherapy (1). Despite significant advances in surgical techniques and radiotherapy strategies, the majority of chordoma patients eventually develop recurrent and/or metastatic disease and ultimately require systemic therapy to control further progression (2). To date, no drugs are approved for the treatment of advanced chordoma and conventional chemotherapy is generally ineffective (1, 2), resulting in a poor prognosis in the advanced disease setting. Research efforts over the past two decades have focused intensively on evaluating drug repurposing opportunities, primarily guided by detection of activated signaling pathways (3–5), focused drug screens (6–8), or anecdotal clinical responses to therapies (9, 10). These investigations inspired several Phase II clinical trials primarily involving multi-kinase inhibition with agents such as imatinib (11), sorafenib (12), lapatinib (13), or everolimus plus imatinib (14), for example, although modest efficacy and low objective response rates were observed in each study. In parallel, efforts to discover novel drug targets indicate that chordoma relies on the lineage-specific transcription factor brachyury (15–17), positioning it as arguably the most attractive – though, as of yet undruggable – target in chordoma.

Over the same time period, the continued growth of genome-guided precision oncology prompted an explosion of drug repurposing efforts for molecularly-defined tumor types – a trend that also extended into the realm of rare cancers. For example, following its approval in chronic myelogenous leukemia, imatinib was successfully repurposed for KIT-mutant gastrointestinal stromal tumors (18), and dabrafenib plus trametinib was repositioned for BRAF V600-mutated anaplastic thyroid cancer (19) after the approval of this combination in non-small cell lung cancer (NSCLC) and melanoma. These and other success stories motivated a series of genomic profiling efforts in chordoma (20–27), with the hope that lifting the veil on chordoma genomes might reveal actionable therapeutic opportunities. Instead, these studies revealed that, similar to most sarcomas and pediatric cancers (28–30), chordoma appears to be characterized by a low and infrequently-actionable mutation burden – with only ~14% of chordomas harboring genomic biomarkers predictive of response to FDA-approved or investigational therapies in other indications (**Table 1**) (31).

**Table 1:** Chordoma mutations reported in genes of potential therapeutic significance.

Gene	Process	Type	Mutation	Reference
PIK3CA	Growth Factor Signaling	Single Nucleotide Variant	–	(21)
		Homozygous Deletion	NA	(21)
		Missense (n=2)	p.E545K	(20), (25)
		Missense	p.M1043I	(20)
		Missense	p.N345S	(25)
PTEN		Missense	p.N48S	(20)
		Frameshift Indel	p.P246fs*8	(25)
		Homozygous Deletion	NA	(25)
		Nonsense	p.R335*	(25)
		Frameshift Indel	–	(21)
		Frameshift Indel	–	(23)
		Missense	p.G251V	(22)
		Nonsense	p.R233*	(22)
PIK3R1		Frameshift Indel	p.M271fs*9	(20)
BRCA2	DNA Damage Repair	Missense	p.A75S	(20)
		Missense	p.R2842C	(25)
		Rearrangement	BRCA2-SPATA13	(25)
		Missense	p.E714A	(26)
		Nonsense	p.G715*	(26)
		Missense	p.I1173F	(26)
		Nonsense	p.C1200*	(26)
		Missense	p.E1593D	(26)
		Missense	p.K1690N	(26)
		Missense	p.E2301G	(26)
		Missense	p.T2337I	(26)
		Missense	p.S2522F	(26)
		Missense	p.N2706S	(26)
		Missense	p.R2784W	(26)
CHEK2		Frameshift Indel	p.T367fs*15	(25)
ATM		Missense	–	(23)
PALB2		Missense	p.S133T	(26)
		Missense (n=2)	p.Q348K	(26)
		Missense	p.S543A	(26)
		Missense	p.V919I	(26)
		Missense	p.I1035V	(26)
		Missense	p.S1165L	(26)
SMARCB1	Chromatin Remodeling	Missense	p.E95K	(24)
		Nonsense	p.E360*	(22)
PBRM1		Single Nucleotide Variant	–	(21)
		Structural Variant (n=5)	–	(21)
		Indel (n=4)	–	(21)
		Missense	p.I555K	(20)
		Frameshift Indel	p.F1007fs*6	(20)
		Nonsense	p.R889*	(20)
		Frameshift Indel	p.F120fs*54	(25)
		Frameshift Indel	p.S383fs*1	(25)
		Homozygous Deletion	NA	(25)
		Frameshift Indel	–	(23)
		Missense	–	(23)
		Nonsense	–	(23)
		Frameshift Indel	–	(23)
		Missense	p.S1315F	(24)
		Nonsense	p.E924*	(22)
ARID1A		Frameshift Indel	p.D641Vfs*8	(20)
		Indel	p.A345_A349del	(25)
		Missense	–	(23)
		Frameshift Indel	–	(23)
		Nonsense	–	(23)
		Nonsense	p.S320*	(22)
ARID1B		Missense	–	(23)
		Missense	p.V602A	(24)
		Indel	p.315_315del	(24)
ARID2		Single Nucleotide Variant	–	(21)
		Homozygous Deletion	NA	(25)
SETD2		Single Nucleotide Variant (n=2)	–	(21)
		Structural Variant	–	(21)
		Frameshift Indel	p.S2253fs*56	(20)
		Frameshift Indel	p.T2338fs*31	(25)
		Missense	–	(23)
		Indel	p.2517_2519del	(24)
		Frameshift Indel	p.P2381fs*	(24)

Mutations denoted with a “-” signify that the mutation type was reported in the associated study without a specific protein alteration call. In such cases, sometimes multiple mutations of the same type were reported, which is signified in parentheses. For alterations classified as single nucleotide or structural variants, no further detail regarding the specific nature of these alterations was provided in the associated study. Alterations colored in red text are existing standard care or investigational biomarkers predictive of response to an FDA-approved or investigational drug in another indication (OncoKB Therapeutic Level 3B; AMP/ASCO/CAP Level C Evidence), and those in blue text may be predictive of response to a drug as supported by compelling biological evidence (OncoKB Therapeutic Level 4; AMP/ASCO/CAP Level D Evidence). The alterations colored red and blue make up 14% of all tumors sequenced across each published profiling study.

Although this observation limits the current impact of traditional genomic profiling on drug repurposing campaigns and precision oncology efforts in chordoma, it does not mean that chordoma is devoid of exploitable alterations *per se* (32). Indeed, genomic profiling studies have identified several potentially actionable alterations based on emerging science – many of which we discuss further below – and validating these therapeutic opportunities may increase the number of advanced-stage chordoma patients that can benefit from genomics-guided precision oncology. Moreover, systematic functional studies in other rare cancers argue that multiple therapeutically actionable vulnerabilities nonetheless exist in the context of a genomically “quiet” background (33–35). In this review, we provide a snapshot of the emerging drug repurposing landscape in chordoma, while highlighting state-of-the-art approaches that can open new windows into chordoma biology to extend our view beyond that provided by genomics. We also discuss opportunities to repurpose lessons learned in other cancers to catalyze the identification of novel therapeutic hypotheses in chordoma. The synthesis of this emerging knowledge may lead to the discovery of new targets and the development of personalized drug repurposing opportunities for chordoma.

## Emerging genomics-guided drug repurposing opportunities in chordoma

**Figure 1:**
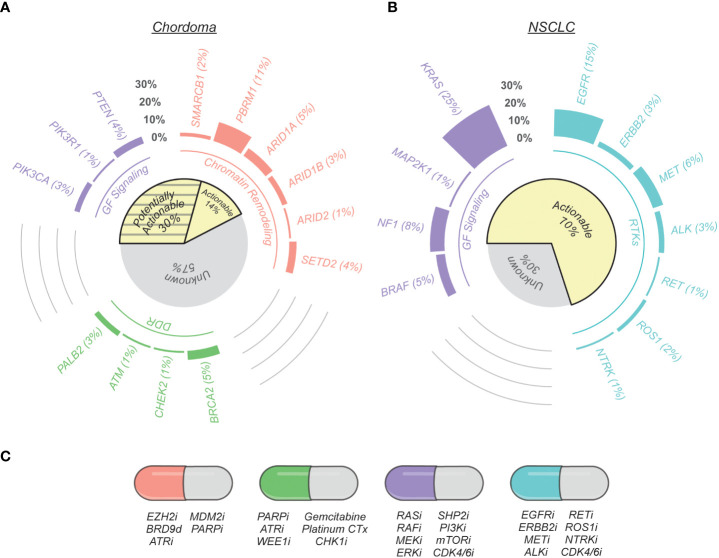
Therapeutically relevant genomic alterations found in chordoma and non-small cell lung cancer (NSCLC). **(A)** Nonsynonymous mutation call data from chordoma tumors were compiled from seven published genomic profiling studies (20–26). Genes are grouped by their protein functionality (chromatin remodeling, DNA damage repair (DDR), growth factor (GF) signaling, and receptor tyrosine kinases (RTKs)) and were selected by on their association with potential therapeutic opportunities based on current scientific literature, as reviewed here. The cohort of tumors analyzed for potentially targetable alterations varied on a per gene basis to account for variation between sequencing techniques and data presentation across studies: SMARCB1 (n = 2 altered/123 total), PBRM1 (22/203), ARID1A (6/123), ARID1B (3/104), ARID2 (2/203), SETD2 (8/203), PTEN (8/179), PIK3CA (6/203), PIK3R1 (1/123), BRCA2 (12/260), CHEK2 (1/123), ATM (1/123), PALB2 (7/260). The subset of actionable gene alterations are existing standard care or investigational biomarkers predictive of response to an FDA-approved or investigational drug in another indication (OncoKB Therapeutic Level 3B; AMP/ASCO/CAP Level C Evidence) or are predictive of response to a drug as supported by compelling biological evidence (OncoKB Therapeutic Level 4; AMP/ASCO/CAP Level D Evidence). Potentially actionable alterations are variants of currently-unknown significance (31). **(B)** Actionable gene alterations in metastatic NSCLC (39). **(C)** Examples of potential therapeutic opportunities indicated by specific gene alterations. The letter “i” signifies an inhibitor, whereas “d” denotes a degrader.

Although ~95-97% of chordomas belong to a single histological subtype, multiple observations suggest that its biology and disease mechanisms are heterogeneous. For example, over half of patients experience disease recurrence following complete tumor resection (2), and exhibit vastly different responses to systemic therapies in the advanced disease setting (36). Additionally, many chordomas are defined by complex genomic rearrangements (20, 37) or recurrent copy number losses (38), whereas other tumors harbor no detectable alterations. This molecular heterogeneity is also reflected in recent chordoma tumor profiling studies, which have utilized next-generation sequencing to identify potentially actionable alterations in chordoma (**Table 1**) (20–23). These studies indicate that only ~14% of chordomas have biomarkers predictive of response to FDA-approved or investigational therapies in other indications. However, several opportunities for molecularly-guided drug repurposing are emerging based on recent scientific advances in chordoma and other cancers, and validation of these therapeutic hypotheses may increase the number of chordoma patients that can benefit from precision oncology (**Figure 1A**).

### Growth factor signaling

In one large cohort (20), PI3K pathway alterations were observed in 16% of cases (*n* = 17/104), indicating an opportunity to explore repurposing of inhibitors targeting PI3K or its downstream effector mTOR. One of the most frequently altered genes in chordoma is PTEN (**Figure 1A**); the resulting potential dependence on PI3Kβ signaling (40) suggests an opportunity to evaluate PI3Kβ inhibitors in chordoma (41). Indeed, a recent preclinical study revealed significant tumor growth inhibition by the pan-PI3K inhibitor buparlisib (BKM120) in patient-derived xenograft models (42). Downstream of PI3K, clinical trials involving mTOR inhibitor combinations have demonstrated modest clinical benefit in chordoma patients, particularly in tumors with mTOR effector activation (14, 43). Intriguingly, chordoma sometimes occurs in patients with tuberous sclerosis complex (44–46), which is characterized by loss of the mTOR negative regulators TSC1/2, further hinting at a role for the PI3K/mTOR pathway in chordoma pathogenesis. Moreover, PI3K and mTOR are regulated by receptor tyrosine kinases (RTKs), of which several appear to be activated in most chordoma tumors (4). Several studies have analyzed the activation state or effects of targeting RTKs including MET (47, 48), IGF1R (49, 50), and the FGFR family (51), though PDGFRβ (5, 10) and EGFR (3, 52) have received the most attention, primarily owing to evidence of some clinical benefit from agents targeting these RTKs (9–11, 13). Since RTK mutations are not frequently seen in chordoma, these receptors are presumably activated through alternative mechanisms such as aberrant growth factor production, which may be directly regulated by brachyury (53). The frequent activation of RTKs observed in chordoma may be related to the role of the notochord in regulating embryonic tissue patterning; in this context RTKs are thought to dictate proliferation and differentiation through the interpretation of morphogen gradients (54, 55). Inhibitors of wild-type EGFR, such as afatinib and cetuximab, have reproducibly shown promising activity against chordoma cell lines (3, 6, 7) and xenograft models (39, 47), which has motivated two Phase II clinical trials (NCT03083678 and NCT05041127). Since these strategies rely on inhibition of wild-type EGFR, it remains to be seen whether skin and gastrointestinal toxicities will limit their efficacy in the clinic (56).

Growth factor signaling drives cell proliferation by upregulation of cyclin D, CDK4/6 activation, and progression through the G1/S cell cycle checkpoint. RTK activation along with frequent loss of the cell cycle tumor suppressor CDKN2A in chordomas (24, 38, 57, 58) has motivated preclinical repurposing studies with CDK4/6 inhibitors (39, 59, 60) and a Phase II trial involving palbociclib in CDKN2A-null chordoma patients (NCT03110744). It remains unclear, however, whether CDKN2A loss is a faithful predictor of sensitivity to CDK4/6 inhibition (61, 62) – possibly because, in addition to p16^INK4A^, CDKN2A encodes p14^ARF^, whose loss results in CDK2 deregulation and compensatory G1/S cell cycle progression. Nevertheless, tumors with co-deletion of the CDKN2A-proximal MTAP gene may present an opportunity for combinations involving CDK4/6 inhibitors and antagonists of the PRMT5 axis (63–65). Notably, CDK4/6 inhibition has been reported to potentiate T cell immunity in several contexts (66, 67), and we discuss opportunities for evaluating CDK4/6 inhibitor combinations in this context further below.

### DNA damage repair

Genomic profiling studies have also revealed potential synthetic lethality strategies in chordoma. Several deleterious alterations have been reported in genes involved in DNA damage repair and response, including BRCA2, CHEK2, PALB2 and ATM (20, 23, 25, 26). In one recent study, a novel defective homologous recombination signature was identified in advanced chordomas that appears to impart a “BRCAness” phenotype and sensitivity to PARP inhibition (22). This strategy is being explored further in a Phase 2 clinical trial combining olaparib plus trabectedin for solid tumors with this defective homologous recombination signature (NCT03127215). Future studies aimed at examining the potential link between DNA damage repair defects and complex genomic rearrangements in chordoma may provide further mechanistic insight into this therapeutic opportunity.

### Chromatin remodeling

Other studies have identified alterations in chromatin remodeling genes such as SETD2 and SWI/SNF complex members SMARCB1, ARID1A, and PBRM1 (20, 21, 24). Notably, biallelic loss of SMARCB1 defines an aggressive, poorly differentiated histopathological subtype of chordoma (<5% of cases) that most commonly afflicts the pediatric patient population (68, 69). Based on the apparent EZH2 dependence bestowed by SWI/SNF alterations (70, 71), a Phase II study is underway to explore repurposing of tazemetostat for SMARCB1-null chordoma (NCT02601950). The presence of SWI/SNF alterations also suggests opportunities for therapeutically exploiting aberrant SWI/SNF function, for example through resulting synthetic lethality with BRD9 antagonists (72–74), inhibitors of DNA repair (75–77), or p53 activation (78). Implementation of functional genomics screens may lead to the discovery of additional chordoma-specific synthetic lethal strategies in this context, which we discuss in more detail below.

An interesting connection appears to exist between SWI/SNF, the Hippo pathway, and brachyury, chordoma’s main Achilles’ heel (79). Hippo transcriptional effectors YAP and TEAD are critical for notochord differentiation during embryonic development (80). Indeed, a YAP/TEAD motif is one of the top brachyury binding sites in chordoma cells (81), and reports have linked brachyury-mediated YAP upregulation to stemness and growth (82) – suggesting convergence between the Hippo and brachyury signaling networks. Intriguingly, SWI/SNF appears to sequester YAP, preventing its association with TEAD and thus antagonizing oncogenic Hippo transcriptional outputs (83). A key role of loss-of-function SWI/SNF alterations in chordoma may therefore be de-sequestration of YAP, which, when augmented by brachyury-mediated upregulation of YAP synthesis and stability, drives Hippo pathway flux to an oncogenic level. These observations suggest opportunities for evaluating an emerging class of TEAD palmitoylation inhibitors (84–86) in chordoma.

## Moving beyond genomics to identify new therapeutic strategies

### Creating a multidimensional map of chordoma cell circuitry

Although genomic profiling studies have informed our understanding of chordoma biology and expanded the list of potentially actionable therapeutic targets, chordoma nevertheless remains largely devoid of the recurring, actionable genomic alterations that define other solid tumors. For example, therapeutic biomarkers guide care for over two-thirds of metastatic NSCLC patients, with response rates to targeted therapies often approaching 70-80%, while chordoma profiling studies indicate ~14% of cases have potentially actionable genomic alterations (**Figure 1**). As highlighted in the previous section, our developing understanding of cancer biology suggests up to an additional ~30% of chordomas might have actionable genomic alterations; nevertheless, a majority of advanced-stage patients lack clear or effective treatment options.

This creates a need to open new windows into chordoma biology that extend our view beyond the “single oncogenic driver” perspective of cancer’s dependencies. To this end, studies across several cancers have revealed new categories of therapeutic targets, called “non-oncogene dependencies”, that mediate epigenetic changes, dysregulated signal transduction, metabolic rewiring, immune evasion, and other hallmarks of cancer (32). Multiple efforts are underway to analyze and integrate data layers derived from different aspects of cell biology, with a view to providing a more detailed molecular-resolution view of chordoma pathogenesis. For example, a recent investigation of methylation signatures in circulating tumor DNA revealed the existence of two distinct epigenetic subtypes in chordoma with prognostic relevance (87). A gene-set enrichment analysis pointed to dysregulated signaling pathways operating within each subtype, uncovering potential therapeutic opportunities that prompt further evaluation in functional studies. The exploration of additional data layers may further elucidate chordoma’s molecular subtypes, including their association with specific therapeutic vulnerabilities, risk of recurrence, and other features of the disease. Such multi-omics studies may also lead to the identification of tumor-specific or lineage-restricted cell surface proteins that can serve as targets for antibody-drug conjugates, bispecific antibodies, chimeric antigen receptor T cells, or other surface antigen-targeted modalities.

### Tumor-host interactions in the tumor microenvironment

In addition to tumor cell intrinsic targets, therapeutic opportunities may exist within the tumor microenvironment, where crosstalk with various immune and stromal cell subsets can profoundly influence chordoma progression and therapy response (88, 89). Studies of the chordoma immune microenvironment to date have focused on the PD-1 axis (90, 91), as well as other potentially important immune checkpoints such as B7-H3 and HHLA2 (92, 93). A recent single-cell transcriptomic analysis of six chordoma tumors identified putative immunosuppressive contributions from regulatory T cells, tumor-associated macrophages, and TGFβ signaling (94). Notably, TGFβ pathway genes are upregulated by brachyury (81). These results point to a repurposing opportunity for antagonists of TGFβ signaling in combination with immune checkpoint blockade (95, 96). Interestingly, a chordoma patient treated with a bifunctional fusion protein targeting TGFβ and PD-L1 experienced late-onset tumor shrinkage in a Phase 1 trial (97). The set of factors that govern antitumor immunity is complex, and more comprehensive phenotyping of the chordoma immune microenvironment – through single-cell sequencing, digital spatial profiling, multispectral immunofluorescence and other approaches – will be important for creating an atlas of the various lineage states in chordoma and revealing therapeutically-reversible defects in the cancer-immunity cycle (98).

### Tumor-host interactions at the physiological level

Other important tumor cell extrinsic features extend beyond the microenvironment, highlighting the need to study chordoma biology at various resolutions – including contributions from host physiology. For example, germline genetics are now understood to play a role in cancer predisposition (99) and tumor immunity (100). Additionally, the gut microbiome impacts immunotherapy efficacy in several solid tumor types (101–103), and recent data indicate that certain dietary habits can modulate the composition of the gut microbiome and influence immunotherapy response (104). Though it remains unclear how these factors contribute to the biology or treatment response of chordoma tumors, some studies are beginning to explore these questions. For example, MD Anderson’s Patient Mosaic initiative aims to collect genetic, immune, and microbiome profiles from thousands of cancer patients to inform treatment strategies. Biospecimens collected from chordoma patients enrolled on the cetuximab Phase II study at MD Anderson will be included in the Patient Mosaic protocol, shedding light on how host (and other tumor extrinsic) factors shape chordoma tumor biology.

### Recent advances in the establishment and availability of chordoma models

The functional validation of new therapeutic targets and strategies resulting from multi-omics studies requires appropriate patient-derived samples and preclinical models. To this end, a variety of chordoma models have been developed by several groups (105, 106). In addition, the Chordoma Foundation has built a tumor biobank of over 500 biospecimens and a model repository currently consisting of 26 cell lines, 12 patient-derived xenograft (PDX) models, and a PBMC-humanized mouse model (www.chordoma.org/research). The majority of these models have been characterized by whole-exome and whole-transcriptome sequencing and will undergo additional multi-omics characterization in the future, with a view to facilitating hypothesis testing through the establishment of models representing the full diversity of chordoma. Moreover, these models are available to the research community, as are in-kind drug testing services offered through the Chordoma Foundation’s Drug Screening Program. As emerging drug repurposing concepts are evaluated in the Drug Screening Program, resulting data are publicly shared, whenever possible (107), to provide justification for further evaluation of the most promising therapeutic opportunities.

## Unbiased functional assays for target discovery and personalized medicine

### Patient-derived models for target discovery and precision oncology

In translational cancer research, PDX models have been the gold standard for preclinical drug testing because they accurately recapitulate features of the patient’s tumor (108, 109); this has motivated the development of over two dozen chordoma PDXs by the Chordoma Foundation and others (47, 105) that represent the anatomical, age, histopathological, and known molecular diversity of chordoma. More recently, patient-derived organoids (PDOs) have generated significant interest as functional models because they provide faithful representations of patient tumors, while improving on initiation time, cost, and efficiency scales compared to PDXs (110). This technology is now being actively explored in chordoma; one recent proof-of-concept study reportedly developed chordoma PDOs from five different patients and screened them against various drugs to nominate personalized repurposing opportunities (111). In other cancer types, PDOs accurately mimic patient drug response (112–114) and have been utilized for personalized therapy (115, 116). The slow growth rate of chordoma tumors provides a large window of opportunity to develop protocols for establishing, validating, screening chordoma PDOs from high-risk or relapsing patients to enable identification of effective drug repurposing opportunities within the timeframe required to make treatment decisions.

### Implementing systematic functional screens to develop new therapeutic hypotheses

Patient avatars like PDXs, PDOs and cell lines also serve as key platforms for target discovery because they allow functional studies capable of revealing or validating non-oncogene dependencies in chordoma. Genome-scale loss-of-function screens in various cancer cell lines have enabled the creation of “dependency maps” (33, 117), and this cutting-edge approach has recently been applied to chordoma to identify selective genetic dependencies (79). Perhaps unsurprisingly, *T* (or *TBXT*), the gene encoding brachyury, appears to be the most selectively essential gene in chordoma. Since brachyury (like most transcription factors) is a challenging drug target, the authors performed a drug repurposing screen and found that inhibitors of CDK9 or CDK7/12/13 (118) downregulate *TBXT* transcription and suppress chordoma cell proliferation. These results have motivated further *in vivo* testing of transcriptional CDK inhibitors, including KB-0742 (119), in the Chordoma Foundation’s Drug Screening Program (120).

Ongoing systematic screening of genetic and chemical vulnerabilities in chordoma is facilitating the development of new therapeutic hypotheses. For example, CDK6 – but not CDK4 – appears to be a genetic essentiality in some chordoma cell lines (79). Outside of their common cell-cycle target RB1, CDK6 possesses a much broader substrate repertoire than does CDK4 (121) – suggesting that one or more non-RB1 targets may be mechanistically linked to chordoma’s CDK6 dependence. One interesting possibility relates to the observation that chordoma cells are sensitive to the lipid hydroperoxidase inhibitor RSL3 (79), which is known to promote ferroptotic cell death via antagonism of GPX4. CDK6 can upregulate glutathione and NADPH via phosphorylation of two glycolytic enzymes (122); depletion of these antioxidants can prime cells for ferroptosis (123). CDK6 may therefore be crucial for maintaining redox homeostasis in chordoma to safeguard against ferroptosis, providing rationale for evaluation of CDK4/6 inhibitors in combination with ferroptosis inducers.

**Figure 2:**
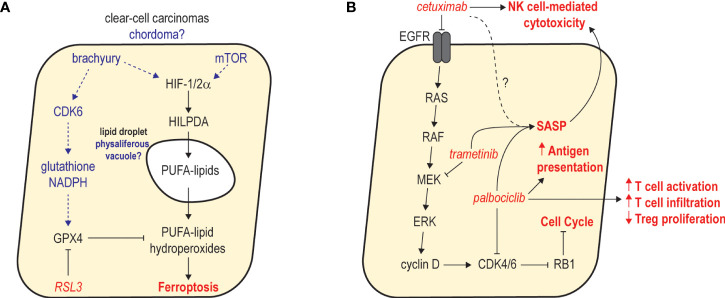
Examples of emerging therapeutic hypotheses in chordoma. **(A)** Potential mechanisms of ferroptosis susceptibility in chordoma and parallels with clear-cell carcinomas. In clear-cell carcinomas, dysregulated HIF-1/2α functions through HILPDA to promote deposition of polyunsaturated fatty acid (PUFA) lipids in intracellular lipid droplets. GPX4 activity counteracts PUFA-lipid oxidation and protects cells from ferroptosis. In chordoma, brachyury and mTOR activity may upregulate HIF-1/2α, resulting in deposition of PUFA lipids in physaliferous vacuoles. CDK6 activity may be critical for antioxidant production to protect chordoma cells against ferroptosis. **(B)** Combined inhibition of MAPK signaling and CDK4/6 activity promotes robust cell cycle arrest and antitumor immunity. Studies in other cancers revealed that combined inhibition of MEK and CDK4/6 with trametinib and palbociclib leads to sustained proliferative arrest and a senescence-associated secretory phenotype (SASP) that promotes NK and T cell immunity. As single agents, cetuximab and palbociclib reportedly augment NK and T cell immunity, respectively. Thus, combining cetuximab with palbociclib may synergistically inhibit tumor growth while stimulating a SASP that augments the activation of NK and T cell-based immunity promoted by each agent.

Chordoma’s apparent CDK6 dependence and potential ferroptosis susceptibility raises intriguing and unexpected parallels with clear-cell carcinomas (124, 125). Histologically, clear-cell renal cell carcinoma (ccRCC) is almost indistinguishable from chordoma, owing to morphological similarities between ccRCC’s characteristic lipid droplets and the physaliferous cells that define conventional chordoma (126). Notably, ferroptosis susceptibility in clear-cell carcinomas has been linked to HIF-1/2α-dependent accumulation of polyunsaturated lipids within the intracellular droplets that give rise to the clear-cell morphology (124). Both brachyury and mTOR are known to upregulate HIF-1α (81, 127–129), suggesting a possible connection between dysregulated hypoxia signaling, physaliferous morphology, and establishment of a ferroptosis-susceptible state in chordoma (**Figure 2A**). Although the precise composition of physaliferous vacuoles remains unclear (130–132), chordoma and ccRCC share additional similarities, including resistance to chemotherapy and modest mutational burdens enriched in chromatin modifier and PI3K/mTOR pathway alterations (133). Collectively, these observations suggest these cancers of different tissue origins share a similar cellular context, and potentially associated therapeutic vulnerabilities – providing opportunities for repurposing lessons learned from a well-studied and common cancer.

### Functional screens to identify synthetic lethalities

Another key implementation of systematic functional screens involves the discovery of synthetic lethal and combination therapy strategies. Loss-of-function screens in large cell line panels have led to identification of new synthetic lethal interactions (117, 134, 135), including PRMT5 dependence in cells with MTAP loss (63, 64). As noted above, the CDKN2A/MTAP locus is frequently deleted in chordoma (20, 21), suggesting an opportunity for repurposing PRMT5 or MAT2A inhibitors (136, 137). Exploiting such synthetic lethalities not only provides an avenue for targeting tumor suppressor loss in cancer, but is a particularly important approach to explore in genomically quiet malignancies. In addition to potential vulnerabilities created by loss of MTAP, SWI/SNF, or homologous recombination repair (as noted above), an intriguing candidate for synthetic lethality screening is LYST – a lysosomal trafficking protein of unknown function that’s lost in 10% of chordomas (20). Functional genomics screens in chordoma cell lines with LYST loss may reveal targetable vulnerabilities created by this unique alteration.

## The next frontiers

### Combination therapy strategies

Preclinical and clinical research has yet to identify a therapy capable of producing frequent responses in chordoma (36), motivating the development of combination strategies aimed at increasing the magnitude and duration of therapeutic benefit. One approach with this goal in mind involves performing unbiased anchor screens, in which genome-wide CRISPR screening is utilized to identify genes whose loss sensitizes cells to a given targeted therapy ‘anchor’ (136). Such genes – if druggable – may serve as attractive targets for combination therapy regimens. A similar approach can also be employed to identify candidate resistance mechanisms – that is, genes whose loss (or gain) reduce sensitivity to the anchor drug.

As one of the most well-validated therapeutic targets in chordoma, inhibitors of wild-type EGFR are arguably the best ‘anchors’ to initially explore in unbiased screens or rational combination studies. Indeed, combination therapy investigations with afatinib (47) or cetuximab (138) have yielded encouraging results. One interesting hypothesis involves combining cetuximab with a CDK4/6 inhibitor (**Figure 2B**). Since CDK4/6-mediated G1/S cell cycle progression is highly dependent on RTK/MAPK signaling, concomitant antagonism of EGFR-mediated cyclin D upregulation and CDK4/6 kinase activity may cause a more complete cell cycle arrest. This effect has been observed in lung (139) and pancreatic cancers (140), where combined MEK and CDK4/6 inhibition induced a profound G1/S arrest, resulting in a senescence-associated secretory phenotype (SASP) that promoted increased NK cell-mediated cytotoxicity and infiltration of CD8+ T cells, respectively. Importantly, as an IgG1 antibody, cetuximab monotherapy appears to promote antibody-dependent NK cell-mediated cytotoxicity in several cancers including chordoma (141). A cetuximab/CDK4/6 inhibitor combination may therefore act synergistically to halt the growth of chordoma tumor cells and provoke a strong NK- and T-cell based antitumor response. As a result, further exploration of this concept may be warranted, particularly once the single-agent activity of cetuximab (NCT05041127) and palbociclib (NCT03110744) in chordoma is benchmarked in the clinic. Notably, similar combination immunotherapy approaches aiming to enhance NK cell-mediated killing have recently been described in chordoma (138), and these strategies were reported to selectively target the reservoir of cancer stem-like cells that promote recurrence and therapy resistance.

### Immunotherapy strategies

Achieving deep and durable responses in chordoma will likely require identification of therapeutic concepts capable of invigorating antitumor immunity. Despite a low tumor mutational burden, a significant proportion of chordomas appear to be characterized by complex genomic rearrangements (20, 37), which may lead to high neoantigen expression. In addition to the examples noted above, documented patient responses to vaccines (142) and PD-1 inhibitors (143–146) provide important proof-of-concept for the use of immunotherapies in chordoma, and prompt evaluation of different immune checkpoints and combinations thereof. For example, strong scientific rationale exists for co-blockade of the PD-1 and TIGIT checkpoints in cancer (147), and a new clinical study enrolling chordoma patients is testing this concept with atezolizumab plus tiragolumab (NCT05286801). Another promising approach involves the cell-surface protein CD24, which is frequently expressed in chordomas and – along with brachyury and low molecular weight cytokeratins – has been used as a diagnostic marker for chordoma in some cases (148). Intriguingly, tumor-derived CD24 was recently identified as a key anti-phagocytic “don’t eat me” signal in other solid tumors (149), making it a promising immunotherapeutic target and prompting evaluation of CD24 blockade in chordoma. The evaluation of immunotherapy combinations in chordoma, such as PD-1 antagonism plus inhibition of TIGIT or TGFβ signaling as noted above, may reinvigorate the tumor-immunity cycle at multiple points. Multi-omics studies of chordoma may be valuable in guiding these efforts and revealing key molecular details governing the chordoma immune microenvironment.

## New drug discovery driving repurposing in reverse

**Figure 3:**
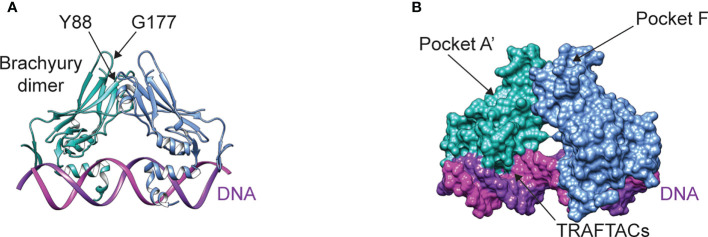
**(A)** Structure of the brachyury dimer bound to DNA (PDB ID 6F58). The alpha carbons of residues Y88 and G177 are separated by a distance of ~9 Å **(B)** Brachyury rendered in surface representation, showing the two main pockets being targeted by open-source drug discovery efforts and the DNA binding interface targeted by TRAFTACs.

Tumorigenesis appears to require three main ingredients: an oncogenic signaling input, deregulation of the signal through tumor suppressor loss (150, 151), and a permissive transcriptional environment for interpretation of oncogenic signaling (152, 153). Lineage-specific transcription factors – such as brachyury in chordoma – are essential for creating a permissive environment (79), and thus represent attractive drug targets. While oncogenic signaling and tumor suppressor loss can be targeted by kinase inhibitors and synthetic lethal strategies, respectively, transcription factors like brachyury are inherently challenging drug targets (**Figure 3A**). However, advances in drug discovery and the development of new targeted protein degradation technologies, such as proteolysis targeting chimeras (PROTACs) and molecular glues, provide opportunities to redefine this paradigm (154). To this end, numerous projects have recently been launched to develop novel compounds that bind brachyury with high affinity, which can either serve as functional inhibitors, molecular glues, or warheads for PROTACS. Notably, an open-source project through the Structural Genomics Consortium is focusing on the development of high-quality probes that bind pockets identified in brachyury crystal structures (**Figure 3B**) to induce industry investment in further brachyury drug discovery.

The development of functional inhibitors is a challenging endeavor, given that brachyury lacks the deep binding pockets commonly associated with enzymatic activity. Yet, transcription factors like brachyury are often involved in multiprotein complexes, pointing to the development of compounds that modulate protein-protein interactions as an attractive strategy. For example, brachyury associates with the histone acetyltransferase p300 using an interface involving amino acid residue Y88 (**Figure 3A**) (155). The proximity of Y88 to residue G177 (**Figure 3A**) is interesting, as a G177D germline variant is strongly associated with chordoma (15). Because residue G177 is on a flexible, solvent-exposed loop, the G177D mutation is unlikely to affect brachyury structure – however this substitution may stabilize intermolecular contacts with p300 or other binding partners, thus modulating brachyury function. Indeed, the interaction between brachyury and p300 appears to regulate histone 3 lysine 27 acetylation (155) – a modification associated with active enhancers. Since association of brachyury with super-enhancers appears to be crucial to its role in chordoma (79, 81), designing compounds that can block or allosterically modulate this protein-protein interaction – for example, by targeting pocket A’ or F (**Figure 3B**) – may represent an attractive therapeutic strategy. Another novel approach to functionally modulating brachyury involves the development of Transcription Factor Targeting Chimeras (TRAFTACs) (156). In contrast to PROTACs, TRAFTACs utilize a transcription factor-specific DNA sequence to achieve target specificity, which is linked to an E3 ligase-recruiting moiety that directs brachyury to the proteasome for degradation.

**Figure 4:**
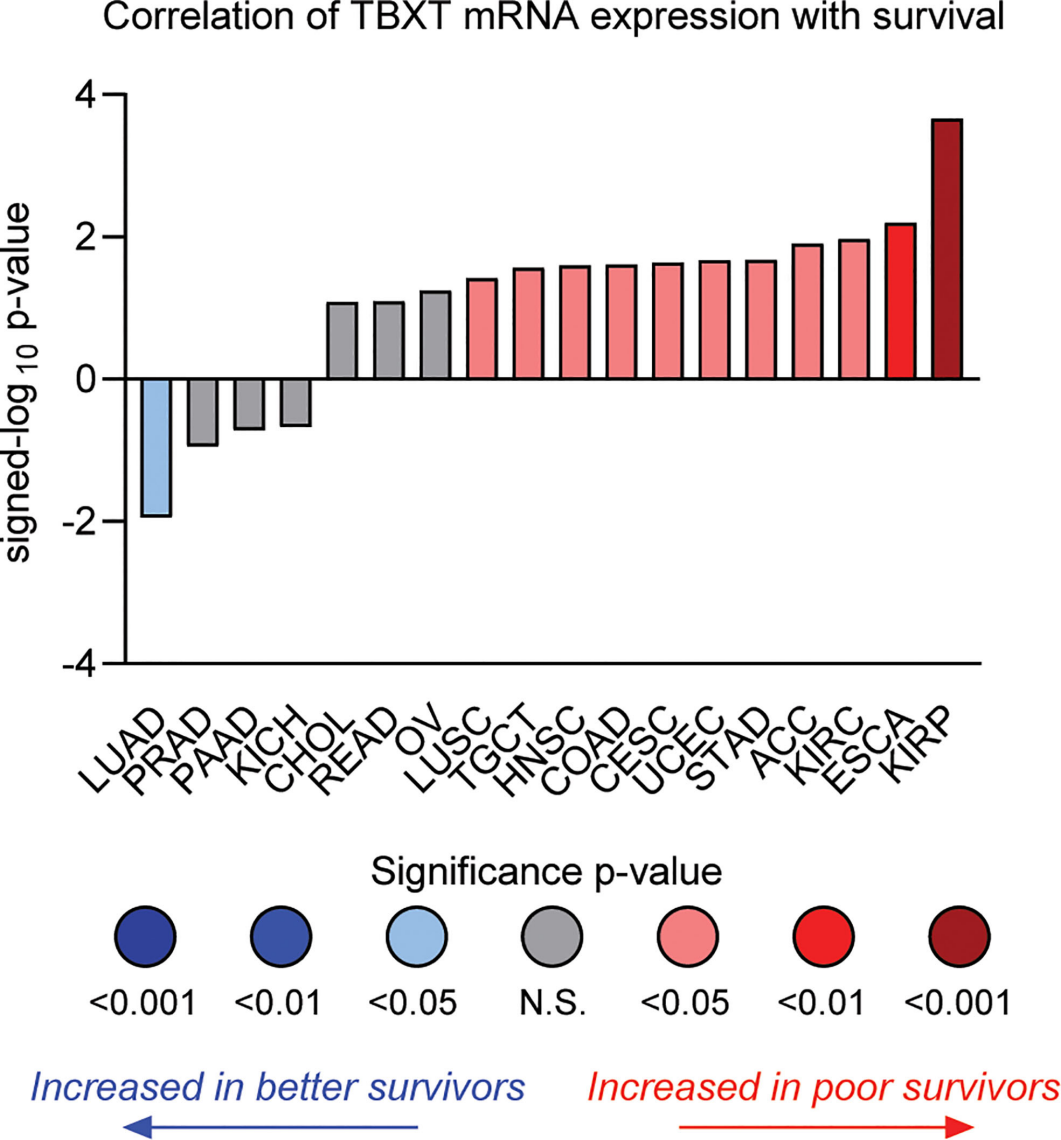
Correlation of *TBXT* (brachyury) mRNA expression with overall survival by tumor type in TCGA datasets. The individual values are sign-corrected log_10_ p-values of correlation. Negative signed-log_10_ p-values (y-axis) indicate that high *TBXT* expression is associated with better survival, whereas positive values indicate high *TBXT* expression predicts poor survival. Cancer types are listed by their respective TCGA study abbreviations (e.g., KIRP, kidney renal papillary cell carcinoma; ESCA, esophageal carcinoma; KIRC, kidney renal clear-cell carcinoma; LUAD, lung adenocarcinoma). The results shown in this figure are in whole or part based upon data generated through the Lumin Bioinformatics Software of Champions Oncology, Inc.

Although new drug discovery in an ultra-rare indication presents numerous challenges, the concept of “reverse” drug repurposing – that is, repurposing drugs initially developed in a rare cancer to more common indications – represents a promising path. Further highlighting the intriguing parallels between chordoma and kidney cancer, brachyury expression is associated with poor survival in ccRCC and papillary RCC (**Figure 4**) (133, 157). Interestingly, expression of CDK6 – an apparent dependency in both chordoma and ccRCC (125), as noted above – appears to be regulated by brachyury (79). Numerous additional studies indicate brachyury is associated with poor prognosis and implicated in driving recurrence, metastasis, and/or resistance to standard of care therapy in several more common cancers including breast (158–161), lung (162–166), and colon (167). Thus, chordoma represents a “pure” and target-rich setting for the initial development of brachyury antagonists, which can then be expanded into larger indications where brachyury plays a role in disease progression.

## Perspective

Even when macroscopic complete resection is achieved using cutting-edge surgical approaches, the majority of chordoma patients experience disease recurrence and are unlikely to be cured (1, 2). At some point, local therapies such as surgery and/or radiation are no longer safe or feasible, and treatment options become limited due to a lack of effective systemic therapies. This has motivated intensive research to identify effective therapeutic strategies in chordoma, but drug repurposing efforts have been hampered by chordoma’s resistance to conventional chemotherapy and a paucity of actionable genomic alterations.

In this review, we highlight several therapeutic hypotheses inspired by developing knowledge of chordoma biology and its parallels with other cancer types. In particular, we focus on emerging therapeutic opportunities based on emerging knowledge linking drug sensitivity to specific biomarkers. Nevertheless, through the lens of genomic sequencing, most chordomas still lack actionable alterations – underscoring the need to implement more sophisticated multi-omics approaches. Indeed, genomics is only one piece of the puzzle; tumor growth is controlled by multiple integrated systems, with each contributing uniquely to chordoma’s biology. Therapeutic opportunities exist within each of these systems, and efforts focused on elucidating and integrating them will provide a fuller view of chordoma’s biology. A key goal of multi-omics profiling efforts will be the identification of molecular subtypes, stratified by risk and therapeutic vulnerabilities, as has been demonstrated in other cancers (168–170).

In parallel, unbiased functional assays, utilizing genome-wide CRISPR or high-throughput drug screening, may reveal non-oncogene dependencies or combination therapy strategies that would otherwise be difficult to detect through multi-omics profiling approaches. The identification of additive or synergistic therapeutic combinations is of particular interest, given the low historical response rates in chordoma (36). In addition to guiding target discovery campaigns, functional assays can provide personalized medicine opportunities. For example, if multi-omics studies identify patients at high risk for recurrence, the ability to establish and profile drug sensitivity of PDXs or PDOs at time of initial surgery may allow nomination of potential therapeutic options upon disease recurrence, as successfully demonstrated recently in breast cancer (116). Due to the intrinsically slow growth of chordoma tumors, such an approach could also be considered at the time of recurrence.

Finally, we highlight how technological advances are opening the door to targeting the transcription factor brachyury, the main Achilles heel of chordoma. The identification of binding pockets on brachyury can serve as target sites for PROTAC warheads or molecular glues, but they may also be functionally important. One such potential site is pocket A’ or F (**Figure 3B**), near the putative p300 interface and residue G177, which is the site of a germline variant strongly associated with chordoma development. If efforts to target brachyury are ultimately successful, these drugs can be repurposed for more common cancers in circumstances where brachyury drives resistance to standard of care therapy.

## Author contributions

All authors listed have made a substantial, direct, and intellectual contribution to the work and approved it for publication.

## Funding

This work was funded by the generous donors to Chordoma Foundation.

## Acknowledgments

We acknowledge and thank our patient community for giving us constant motivation to help identify and develop better treatments for chordoma. We are also grateful for our generous donors who provide the necessary funding – and to our colleagues at Chordoma Foundation and the research community for their efforts – to work towards this goal. Finally, we thank Champions Oncology for their assistance with figure generation.

## Conflict of interest

The authors declare that the research was conducted in the absence of any commercial or financial relationships that could be construed as a potential conflict of interest.

## Publisher’s note

All claims expressed in this article are solely those of the authors and do not necessarily represent those of their affiliated organizations, or those of the publisher, the editors and the reviewers. Any product that may be evaluated in this article, or claim that may be made by its manufacturer, is not guaranteed or endorsed by the publisher.
